# Relationship between miRNA and ferroptosis in tumors

**DOI:** 10.3389/fphar.2022.977062

**Published:** 2022-11-04

**Authors:** Shang-Ming Dai, Feng-Jiao Li, Hui-Zhi Long, Zi-Wei Zhou, Hong-Yu Luo, Shuo-Guo Xu, Li-Chen Gao

**Affiliations:** ^1^ Department of Pharmacy, Cancer Institute, Phase I Clinical Trial Centre, Changsha Central Hospital Affiliated to University of South China, School of Pharmacy, University of South China, Changsha, China; ^2^ Hunan Provincial Key Laboratory of Tumor Microenvironment Responsive Drug Research, Hengyang, China

**Keywords:** ferroptosis, microRNA, tumor, mechanism, proliferation, invasion, metastasis, sensitivity of treatment

## Abstract

Malignant tumor is a major killer that seriously endangers human health. At present, the methods of treating tumors include surgical resection, chemotherapy, radiotherapy and immunotherapy. However, the survival rate of patients is still very low due to the complicated mechanism of tumor occurrence and development and high recurrence rate. Individualized treatment will be the main direction of tumor treatment in the future. Because only by understanding the molecular mechanism of tumor development and differentially expressed genes can we carry out accurate treatment and improve the therapeutic effect. MicroRNA (miRNA) is a kind of small non coding RNA, which regulates gene expression at mRNA level and plays a key role in tumor regulation. Ferroptosis is a kind of programmed death caused by iron dependent lipid peroxidation, which is different from apoptosis, necrosis and other cell death modes. Now it has been found that ferroptosis plays an important role in the occurrence and development of tumors and drug resistance. More and more studies have found that miRNAs can regulate tumor development and drug resistance through ferroptosis. Therefore, in this review, the mechanism of ferroptosis is briefly outlined, and the relationship between miRNAs and ferroptosis in tumors is reviewed.

## Introduction

Malignant tumors are a major killer of human life and health. Currently, there are surgical resection, chemotherapy, radiotherapy, immunotherapy and other treatment methods (Dasari and Tchounwou, 2014; Yang, 2015; Bradley and Mendenhall, 2018), but the patient mortality is still very high. In particular, some malignant tumors have developed resistance to traditional treatment methods, making the treatment effect poor (Szakács et al., 2006; Du and Shim, 2016; Sharma et al., 2017; O'Donnell et al., 2019). Therefore, the new way of tumor cell death is of great significance to the treatment of tumor and to improve the sensitivity of traditional therapy.

The concept of ferroptosis was first defined by Dixon in 2012. It is a new programmed cell death mode that is different from apoptosis and necrosis in morphology, biochemistry and genetics. Its important feature is iron dependent lipid peroxidation (Dixon et al., 2012; Proneth and Conrad, 2019; Yan and Zhang, 2019; Li Y. et al., 2020). Before the concept of ferroptosis was put forward, it was found that erastin was lethal to human foreskin fibroblasts expressing mutant Ras oncogene in 2003 (Dolma et al., 2003). However, no target of cell death induced by erastin was found in subsequent studies (Yagoda et al., 2007). Until 2012, this cell death mode was officially named ferroptosis, and it was found that erastin blocked the uptake of cystine of cells by inhibiting the cystine/glutamate antiporter (system xc^−^), so that the reducing substances were exhausted, resulting in cell death (Dixon et al., 2012). At present, ferroptosis has been found to be involved in the development of neurodegenerative diseases (Hambright et al., 2017), ischemia-reperfusion injury (Li et al., 2019), malignant tumors (Zhang et al., 2021b; Sun J. et al., 2021), and other diseases.

MicroRNA (miRNA) is a kind of small non coding RNA, which can directly combine with the 3′- untranslated region (3' - UTR) of the target mRNA to inhibit the translation of the target mRNA, thus regulating a series of cellular biological processes (Bartel, 2004; Esteller, 2011; Rupaimoole and Slack, 2017). It has been found that miRNA can conduct through the ferroptosis signal pathway, and then play a crucial role in the occurrence and development of tumors (Cai et al., 2022; Pan et al., 2022; Zhang N. et al., 2022).

This article mainly reviews the recent research on microRNA targeting ferroptosis signaling pathway in tumors, and provides new ideas for tumor diagnosis, treatment and improving treatment sensitivity.

## MicroRNA targeting iron metabolism

Iron is an indispensable substance for cells to maintain physiological and biochemical functions. Under normal conditions, iron is strictly regulated in cells and maintains a relatively stable level (Aisen et al., 2001; Zhang, 2014; Morales and Xue, 2021). Extracellular Fe^3+^ can be endocytosed by binding with transferrin (TF) and then binding with transferrin receptor 1 (TFR1, TFRC) on the cell membrane to form endosomes, thus transferring extracellular Fe^3+^ into cells (El Hout et al., 2018). Fe^3+^ in the acidic endosomes is reduced to Fe^2+^ under the action of prostate six transmembrane antigen protein 3 (STEAP3), and then transferred to the cytoplasm by divalent metal ion transporter 1 (DMT1, SLC11A2) or zinc transporter 8/14 (ZIP8/14), and introduced into the labile iron pool (LIP) (Bogdan et al., 2016). In addition, DMT1 and ZIP8/14 can also directly transport extracellular free Fe^2+^ to intracellular LIP (Liuzzi et al., 2006; Wang et al., 2012). Excessive Fe^2+^ in lip can produce a large amount of reactive oxygen species (ROS) through Fenton reaction, causing lipid peroxidation and cell death (Stockwell et al., 2017). While poly (RC) binding protein 1 (PCBP1) and poly (RC) binding protein 2 (PCBP2) can load Fe^2+^, bind it to ferritin and store it in the form of Fe^3+^, so as to reduce the content of free iron in cells and avoid oxidative damage to cells (Shi et al., 2008; Leidgens et al., 2013). In the case of intracellular iron deficiency, ferritin can also be transported to autophagosome under the action of nuclear receptor coactivator 4 (NCOA4), and ferritin will release Fe^3+^ again due to autophagy; When cells are not iron deficient, ferritin is degraded through non autophagy (Asano et al., 2011; Mancias et al., 2014). Iron response element binding protein 2 (IREB2) is an mRNA binding protein that can bind to iron response element (IRE) on mRNA and is the main regulator of iron metabolism related genes. IREB2 can down regulate the expression of ferritin (FTH1 and FTL), ferroportin (FPN, SLC40A1), up regulate the expression of TFRC and SLC11A2, and finally increase the concentration of intracellular free iron (Ooko et al., 2015; Cooper et al., 2019; Chen X.et al., 2021). Ferroportin (FPN, SLC40A1) is a key protein that can transport Fe^3+^ to the outside of cells and plays a very important role in maintaining stable iron content in cells (Ganz, 2005). In addition, prominin2, a protein encoded by PROM2 gene, was found to drive the production of multivesicular bodies (MVB). MVB ingests ferritin in cells and fuses with plasma membrane to expel ferritin out of cells in the form of secretory body (Belavgeni et al., 2019).

In HT-29 and HCT-116 human colorectal cancer (CRC) cells treated with ferroptosis inducers erastin (SLC7A11 inhibitor) and RSL3 (GPX4 inhibitor), the silencing of miR-545 could increase the levels of intracellular ferroptosis biomarkers malondialdehyde (MDA), reactive oxygen species (ROS) and Fe^2+^ and reduce the viability of the cells, while the overexpression of miR-545 was the opposite. In mechanism, miR-545 inhibits the progress of ferroptosis by inhibiting the expression of TF, and promotes the viability of CRC cells. When Erastin-treated CRC cells with miR-545 knockdown were injected subcutaneously into nude mice, Erastin reduced tumor volume, and inhibition of miR-545 further reduced tumor volume (Zheng et al., 2021). In addition, miR-19a has also been found to promote the proliferation, invasion and metastasis of CRC cells, and its mechanism is that miR-19a inhibits the expression of IREB2, thereby inhibiting the ferroptosis process of cells (Fan et al., 2022).

MiR-4735-3p was found to be low expressed in clear cell renal cell carcinoma (ccRCC). The inhibition of miR-4735-3p reduced the level of lipid peroxidation and Fe^2+^ in ccRCC cells and inhibited the progression of ferroptosis. Further research found that miR-4735-3p can promote the ferroptosis process of ccRCC cells by directly targeting SLC40A1, and ultimately inhibit the development of tumors (Zhu et al., 2022).

In the process of exploring the effect of long-chain noncoding RNA (lncRNA) RP11-89 on the development of bladder cancer, it was found that RP11-89 could act as a “sponge” to adsorb miR-129-5p and up regulate PROM2. The up regulation of PROM2 in cells can promote the formation of MVB, the output of iron and the decrease of mitochondria, which weakens the effect of ferroptosis. RP11-89 promotes cell proliferation, migration and tumorigenesis through miR-129-5p/PROM2 axis, and inhibits cell cycle arrest. This suggests that RP11-89 and mi-129-5p can be used as potential targets for the treatment of bladder cancer (Luo W. et al., 2021).

Curcumin is a natural product extracted from Curcuma wenyujin. It can inhibit the proliferation of lung cancer cells and induce cell death. The main form of death is ferroptosis. Curcumin can significantly down regulate the expression of long-chain non coding RNA H19 (lncRNA H19) in lung cancer cells. LncRNA H19, as a competitive endogenous RNA (ceRNA), binds to miR-19b-3p and can enhance the transcription of FTH1. Therefore, curcumin blocks the progression of lung cancer by inhibiting the lncRNA H19/miR-19b-3p/FTH1 axis to promote the ferroptosis process (Zhang R. et al., 2022).

In a word, iron plays a very important role in the physiological process of cells. Under normal conditions, iron maintains a relatively stable level. Lack of iron will lead to the failure of cell function, while excessive iron will lead to cell oxidative stress and promote cell ferroptosis.

## MicroRNA targeting GSH synthesis pathway

Cystine/glutamate antiporter (system xc^−^) is composed of solute carrier family member 7A11 (SLC7A11) and solute carrier family member 3A2 (SLC3A2) connected by disulfide bonds. Its main function is to promote the exchange of cystine outside the membrane and glutamate inside the membrane (Sato et al., 1999; Cao and Dixon, 2016). Glutamic acid can be produced by glutamine degradation under the catalysis of glutaminase (GLS, including GLS1 and GLS2), in which GLS2 is the target of tumor suppressor gene P53 (Gao et al., 2015; Kang et al., 2019). Cystine transported into cells is reduced to cysteine under the action of thioredoxin reductase 1 (TXNRD1), and then with glutamic acid and glycine, glutathione (GSH) is generated in two steps under the catalysis of glutamic acid cysteine ligase (GCL) and glutathione synthetase (GSS) (Liang et al., 2019; Jiang et al., 2021). GSH is a very important antioxidant in cells. It can reduce lipid peroxide under the catalysis of glutathione peroxidase 4 (GPX4) to avoid oxidative damage to cells. If erastin is used to inhibit the activity of system xc^−^, the GSH synthesis path will be blocked, and the intracellular lipid peroxide cannot be cleared, resulting in cell ferroptosis (Dixon et al., 2014).

MiR-375 is a multifunctional miRNA that participates in islet development, insulin secretion and cell proliferation (Li, 2014). MiR-375 was found to be down-regulated in gastric cancer (GC) stem cells. Up regulation of miR-375 can significantly inhibit the number of GC stem cells. The mechanism is that miR-375 triggers cell ferroptosis by directly targeting SLC7A11. When GC cells were subcutaneously injected into nude mice, miR-375 was able to suppress tumor volume and decreased stem cell frequency. When nude mice were injected with only a small number of cells, miR-375 might prevent the mice from forming tumors. These results were rescued by overexpression of SLC7A11. (Ni et al., 2021). Therefore, miR-375/SLC7A11 axis may be a target for inducing ferroptosis in GC stem cells.

MiR-5096 can inhibit the proliferation and invasion of breast cancer cells and induce cell death. Overexpression of miR-5096 causes the increase of iron content, ROS, hydroxyl radical, lipid peroxide and the decrease of GSH in breast cancer cells. These characteristics indicate that its effect is related to ferroptosis. Through the study of its mechanism, it is found that miR-5096 induces ferroptosis in breast cancer cells by inhibiting the activity of SLC7A11 (Yadav et al., 2021).

Lidocaine, a local anesthetic commonly used in clinic, was found to inhibit the proliferation, invasion and migration of ovarian and breast cancer cells, and increase intracellular Fe^2+^ and ROS. Further studies showed that lidocaine could down regulate the expression of SLC7A11 by increasing miR-382-5p. Inhibition of miR-382-5p can block lidocaine induced ferroptosis in ovarian and breast cancer cells. Therefore, miR-382-5p may be a good target for inducing ferroptosis in ovarian and breast cancer cells (Sun D. et al., 2021). In addition, levobupivacaine, another local anesthetic, was found to inhibit the growth of gastric cancer cells by inducing ferroptosis of gastric cancer cells through miR-489-3p/SLC7A11 axis (Mao et al., 2021).

SLC7A11 is a very important regulatory molecule in the ferroptosis signaling pathway. At present, a large number of miRNAs have been found to regulate the ferroptosis process of tumor cells by targeting SLC7A11. For example, miR-545-3p/SLC7A11 axis in thyroid cancer (Wang H.H. et al., 2021), miR-125b-5p/SLC7A11 axis and miR-34c-3p/SLC7A11 axis in human oral squamous cell carcinoma (Yu et al., 2021; Sun et al., 2022), circEPSTI1/miR-375, miR-409-3p, miR-515-5p/SLC7A11 axis in cervical cancer (Wu et al., 2021), circ0097009/miR-1261/SLC7A11 axis in hepatocellular carcinoma (Lyu et al., 2021), c-Myc/miR-25-3p/SLC7A11 axis in prostate cancer (Jiang et al., 2022)and lncRNA SLC16A1-AS1/miR-143-3p/SLC7A11 axis in renal cell carcinoma (Li et al., 2022), these signaling pathways involve miRNAs targeting SLC7A11.

In conclusion, SLC7A11 is the main component of system xc^−^ to play a role. The inhibition of SLC7A11 will block the entry of cystine into the cells, block the supply of GSH synthetic raw materials and inhibit the activity of GPX4. The antioxidant system of the cells is damaged, resulting in the failure to remove lipid peroxides in time, and eventually ferroptosis.

## MicroRNA targeting GPX4

Glutathione peroxidase 4 (GPX4), a selenoprotein, is the central regulator of ferroptosis regulation pathway. Selenocysteine residue is the most important active site in GPX4. If it is mutated, it will reduce the activity of GPX4 by 90% (Ingold et al., 2018). Selenium is the raw material for the synthesis of selenocysteine, is the essential element for the formation of GPX4 active site, and plays an important role in the occurrence and development of ferroptosis. The isopentenyl pyrophosphate (IPP) produced by MVA pathway is crucial for the maturation of selenocysteine-tRNA, which also explains the phenomenon that statins cause the decreased expression of GPX4 and the occurrence of ferroptosis by inhibiting MVA pathway (Viswanathan et al., 2017). In addition, cysteine cannot replace selenocysteine to play a role because of the difference in pKa between the two (Borchert et al., 2018). At physiological PH, selenocysteine is more likely to exist in ionic state, which is necessary for catalytic function. The catalytic process of GPX4 is completed in two stages. In the first stage, the selenocysteine residue of the active site reduces the lipid peroxide (PE-AA-OOH and PE-AdA-OOH) to a non-toxic phospholipid alcohol (PE-AA-OH and PE-AdA-OH), while the selenocysteine residue itself is oxidized. In the second stage, the oxidized selenocysteine residues are restored to activity by reduction with 2 molecules of GSH, and GSH is oxidized to GSSG (Forcina and Dixon, 2019). Blocking GSH synthesis or excessive consumption of GSH will make the GPX4 active site unable to recover, which will inhibit GPX4 activity and cause ferroptosis of cells (Yuan et al., 2021). In addition, the use of GPX4 inhibitors (such as RSL3) can directly inhibit GPX4, so that lipid peroxides cannot be effectively cleared, and ultimately lead to ferroptosis of cells (Yang and Stockwell, 2008).

MiR-15a-3p plays an important role in the regulation of various cancers. It is found that miR-15a-3p participates in the ferroptosis process of colorectal cancer, can directly bind to the 3′-UTR of GPX4 and inhibit its activity, resulting in the increase of intracellular ROS, intracellular Fe^2+^ level and MDA (Liu et al., 2022). In addition, it was found that miR-539 was low expressed in colorectal cancer, while tumor necrosis factor (TNF)- α Induced protein 8 (TNFAIP8/TIP8) is highly expressed in colorectal cancer. TIP can promote the proliferation, migration and angiogenesis of colorectal cancer. After exploring the mechanism, it was found that miR-539 can regulate the expression of TIP and indirectly down regulate the expression of GPX4 by activating SAPK/JNK pathway, promoting the process of ferroptosis and inhibiting colorectal cancer cells (Yang Y. et al., 2021). Therefore, miR-15a-3p and miR-539 can be used as potential targets to induce ferroptosis in colorectal cancer cells.

MiR-324-3p was found to be significantly underexpressed in A549 cisplatin resistant lung adenocarcinoma cells. Overexpression of miR-324-3p could reverse the cisplatin resistance of the cells. It was found that miR-324-3p could directly act on GPX4 and inhibit its expression, while overexpression of GPX4 reversed the cisplatin sensitization effect of miR-324-3p on lung adenocarcinoma cells. The GPX4 inhibitor RSL3 has the same effect as the up regulation of miR-324-3p in lung adenocarcinoma cells. Therefore, miR-324-3p/GPX4 axis can be used as a good target to increase cisplatin sensitivity of lung adenocarcinoma cells (Deng et al., 2021).

Metformin is a commonly used hypoglycemic drug in clinic. Many studies have found that it has anticancer activity. In one study, metformin could up regulate the expression of miR-324-3p and induce ferroptosis of MDA-MB-231 breast cancer cells. In its target for ferroptosis, it was found that miR-324-3p could directly bind to the 3′-UTR of GPX4, resulting in the down regulation of GPX4, which led to ferroptosis of cells (Hou et al., 2021). Ketamine, a clinical intravenous anesthetic, was found to inhibit the proliferation of liver cancer cells *in vivo* and *in vitro*, and induce ferroptosis. The expression of lncPVT1 and GPX4 were decreased. Further studies showed that lncPVT1 could directly interact with miR-214-3p and block its adsorption on GPX4. Knockout of lncPVT1 can cause ferroptosis in cells, and inhibition of miR-214-3p or overexpression of GPX4 can reverse this process. Ketamine induced ferroptosis can also be reversed by inhibiting miR-214-3p or overexpression of GPX4. That is, ketamine regulates the ferroptosis process of liver cancer cells through the lncPVT1/miR-214-3p/GPX4 axis (He et al., 2021).

MiR-1287-5p/GPX4 axis is involved in regulating the proliferation activity and ferroptosis of human osteosarcoma cells, and is related to the sensitivity of cells to cisplatin (Xu Z. et al., 2021). In addition, circKIF4A/miR-1231/GPX4 axis in papillary thyroid cancer (Chen W.et al., 2021), miR-15a/GPX4 axis in prostate cancer (Xu et al., 2022), circIL4R/miR-541-3p/GPX4 axis in hepatocellular carcinoma (Xu et al., 2020) and circDTL/miR-1287-5p/GPX4 axis in non-small cell lung cancer (NSCLC) (Shanshan et al., 2021) also regulate the process of ferroptosis in corresponding tumors.

In conclusion, GPX4 is the most important antioxidant factor in the ferroptosis regulatory network. If GPX4 is inhibited, it will lead to the destruction of the antioxidant system, and the lipid peroxides in cells cannot be removed in time, resulting in ferroptosis.

## MicroRNA targeting lipid metabolism

Polyunsaturated fatty acid (PUFA) is an important component of cell membrane and plays an important role in biological function regulation (Gill and Valivety, 1997). PUFA is one of the main targets of intracellular reactive oxygen species (ROS) attack, and its diallyl C-H is vulnerable to attack, leading to lipid peroxidation and cell death (Porter et al., 1979; Yin et al., 2011). Polyunsaturated fatty acids are more prone to lipid peroxidation than monounsaturated fatty acids (MUFA). Providing exogenous PUFA to cells can increase the sensitivity of cells to ferroptosis, while adding exogenous MUFA is the opposite (Yang et al., 2016; Magtanong et al., 2019). In the process of ferroptosis, arachidonic acid (AA) and adrenal acid (AdA) are the two most important PUFAs involved in lipid peroxidation (Kagan et al., 2017). AA and AdA can be esterified to AA-CoA and AdA-CoA by acetyl CoA under the action of long chain acyl CoA synthetase 4 (ACSL4). Then, under the catalysis of lysophosphatidylcholinyltransferase 3 (LPCAT3), it can combine with phosphatidylethanolamine (PE) on the cell membrane to form PE-AA and PE-AdA, so as to transfer these two PUFAs to the cell membrane. Then, PE-AA and PE-AdA on the membrane can be oxidized under the action of Lipoxygenase (LOX) to form two lipid peroxides, PE-AA- OOH and PE-AdA- OOH (Dixon et al., 2015; Yuan et al., 2016; Doll et al., 2017; Kagan et al., 2017; Kuang et al., 2020). Lipid peroxide can change the structure of lipid membrane, resulting in the increase of biofilm curvature, the thinning of membrane thickness and the change of cell permeability, which is an important cause of cell death (Gaschler and Stockwell, 2017; Feng and Stockwell, 2018). In addition, the decomposition of lipid peroxidation products can produce substances such as malondialdehyde (MDA) and 4-hydroxy-nonanal (4-HNEs), which can combine with biological macromolecules such as nucleic acids and proteins to produce cytotoxicity and cause cell death (Ayala et al., 2014).

Exosomes belong to the extracellular vesicle (EV) family, which can transfer proteins, lipids, lncRNAs, circRNAs and miRNAs, and act as an intercellular information transmitter (Théry et al., 2002; He et al., 2018). Exosomes produced by cancer associated fibroblasts (CAFs) in the tumor microenvironment (TME) can promote tumor proliferation, metastasis and increase drug resistance of tumor cells (Li et al., 2018; Dong et al., 2020). The exosome miR-522 secreted by CAF was found to be an inhibitor of arachidonic acid lipoxygenase 15 (ALOX15) in GC, and ALOX15 is the main enzyme that catalyzes the production of lipid peroxide in GC cells. Therefore, miR-522 inhibits ferroptosis by inhibiting the activity of ALOX15, preventing lipid peroxidation of cells. In addition, it was also found that heterogeneous nuclear ribonucleoprotein A1 (hnRNPA1) can promote the transfer of miR-522 to exosomes, and ubiquitin specific protease 7 (USP7) can stabilize hnRNPA1 through deubiquitination. When miR-522 knockdown CAF cells and GC cells were mixed and injected subcutaneously into nude mice, miR-522 in exosomes was reduced. The levels of ALOX15 and lipid ROS in tumor tissues were increased, and the tumor volume was significantly decreased (Zhang et al., 2020).

In the treatment of cancer, the resistance to radiotherapy is one of the thorny problems. Clinically relevant anti-radiation (CRR) cells have strong anti-radiation ability, and also have resistance to anticancer drugs and hydrogen peroxide. High expression of miR-7-5p was found in CRR cells, and knockout of miR-7-5p could lead to radiosensitivity. ROS, mitochondrial membrane potential and intracellular Fe^2+^ concentration of CRR cells with miR-7-5p knockout were significantly increased, while ferritin expression was down regulated and ALOX12 expression was up regulated. This means that miR-7-5p regulates the sensitivity of cells to radiation through the ferroptosis pathway, and ALOX12 is the downstream target of miR-7-5p (Tomita et al., 2021).

Significant changes in intracellular lipid metabolism were found in glioblastoma (GBM), in which the expression of ALOXE3 was significantly down regulated. ALOXE3 knockout can promote the growth of GBM cells and make GBM cells resistant to P53-SLC7A11 dependent ferroptosis. In mechanism, miR-18a can directly bind to ALOXE3 and inhibit its expression and function. In addition, ALOXE3 silencing can promote GMB cells to secrete 12-hydroxyeicosapentaenoic acid (12-HETE), and 12-HETE activates Gs protein coupled receptor (GsPCR) -PI3K-Akt signaling pathway through autocrine mode, promoting the migration of GBM cells. Therefore, miR-18a/ALOXE3 axis regulates the ferroptosis and metastasis of GBM cells (Yang X. et al., 2021).

Sorafenib is a multi-target inhibitor in the treatment of tumors and can be used as a first-line drug in the treatment of advanced hepatocellular carcinoma (HCC). However, the emergence of drug resistance makes the treatment effect poor. The study found that the expression of miR-23a-3p was upregulated in sorafenib insensitive HCC patients compared with sorafenib sensitive HCC patients. Lu et al. constructed HCC tumor bearing mice, and the tumors in the control group continued to grow. However, in mice treated with sorafenib orally for a long time, the tumor volume decreased briefly and then grew again rapidly, which means the emergence of drug resistance. HCC cells were isolated from drug-resistant mice and the IC_50_ value of the cells to sorafenib was significantly increased. More notably, the expression of miR-23a-3p in sorafenib resistant cells was more than 10 times higher than that in parental cells. This means that the upregulation of miR-23a-3p may be the reason why HCC cells acquire sorafenib resistance. In-depth studies found that the upregulation of miR-23a-3p was related to the activation of the upstream gene ETS1. More importantly, the reason why miR-23a-3p makes HCC cells sorafenib resistant is that it down-regulates ACSL4 expression, increases GPX4 expression, reduces Fe^2+^ and lipid ROS content, and puts cells in a high antioxidant state. This attenuates the effect of sorafenib on induction of ferroptosis in HCC. Intervention of the expression of miR-23a-3p may be one of the good ways to sensitize HCC patients to sorafenib again (Lu Y. et al., 2022).

Ma et al. (2021) found that in the detection of 38 pairs of ovarian cancer and its adjacent tissues, the expression of ACSL4 was up-regulated in ovarian cancer and correlated with poor prognosis. But the upregulation of ACSL4 increased the effect of erastin and RSL3 induced ferroptosis in ovarian cancer cells. miR-424-5p inhibitor can promote the ferroptosis of ovarian cancer cells induced by erastin and RSL3, which is related to directly targeting ACSL4 and increasing its expression. This means that although inhibiting miR-424-5p may promote the progression of ovarian cancer, it may also become a method to sensitize ferroptosis in ovarian cancer.

Ou et al. (2022) found that the expression of circular RNA circLMO1 was significantly down-regulated in cervical cancer. Overexpression of circLMO1 can inhibit the proliferation of cervical cancer cells, which can be blocked by ferroptosis inhibitor and apoptosis inhibitor, but not necrosis inhibitor and pyroptosis inhibitor. This indicates that circLMO1 can cause cell death through ferroptosis and apoptosis. In cervical cancer transplanted mice, circLMO1 overexpression significantly suppressed tumor volume. In subsequent studies, the mechanism that circLMO1 can induce ferroptosis in cervical cancer in a miR-4291/ACSL4 dependent manner was determined. Although this may only be one of the pathways, it also provides a potential target for clinical treatment.

Bao et al. (2021) found that miR-670-3p was significantly up-regulated in glioblastoma, but down-regulated under the action of erastin or RSL3. MiR-670-3p inhibitor was able to target ACSL4 to inhibit the growth of glioblastoma and promote ferroptosis of glioblastoma. Silencing ACSL4 almost completely blocked these effects. This suggests that ACSL4 is required for miR-670-3p to regulate ferroptosis and cell growth in glioblastoma. This may be a good target for clinical treatment of glioblastoma, which needs further research.

## MicroRNA targeting FSP1

In the process of ferroptosis, ACSL4 is considered to be an important promoter gene in the regulation of ferroptosis, and the expression level of ACSL4 plays a decisive role in the sensitivity of ferroptosis (Doll et al., 2017). However, in some tumor cells with high expression of ACSL4, even the absence of GPX4 could not cause cell ferroptosis, which indicates that there is an antioxidant system independent of GSH/GPX4. Mitochondrial apoptosis inducing factor 2 (AIFM2) was found to inhibit the occurrence of ferroptosis and was not affected by GSH content and GPX4 activity. In order to characterize the role of AIFM2 in ferroptosis, it was renamed as ferroptosis suppressor protein 1 (FSP1) (Doll et al., 2019). FSP1 functions as NAD (P) H-dependent CoQ oxidoreductase *in vitro* (Marshall et al., 2005). The N-terminal of FSP1 can undergo myristoylation, which can mediate the aggregation of FSP1 to the plasma membrane and play the function of oxidoreductase on the plasma membrane. FSP1 catalyzes the transformation of oxidized CoQ10 (ubiquinone) to reduced CoQ10 (ubiquinol). Reduced CoQ10 acts as an antioxidant to capture lipid free radicals and block the further expansion of lipid peroxidation of the plasma membrane (Li J. et al., 2020; Tang and Kroemer, 2020). CoQ10 is the main effector in FSP1 pathway and can be synthesized by mevalonate (MVA) pathway. In MVA pathway, acetyl CoA molecule generates 3-hydroxy-3-methylglutaric acid monoacyl CoA (HMG CoA) in two steps under the action of enzyme, and then reduces to MVA under the action of HMG CoA reductase (HMGCR). MVA can produce IPP through a series of enzymatic reactions. IPP can be used as the basic unit for the synthesis of downstream products. IPP generates farnesyl pyrophosphate (FPP) and geranylgeranyl pyrophosphate (GGPP) through various enzymatic steps, and is finally catalyzed to CoQ10 (Miziorko, 2011; Mullen et al., 2016; Yan et al., 2021). The antioxidant system FSP1/CoQ10/NAD(P) H plays a role independently of GSH/GPX4 system, and they play a synergistic role in the process of resisting lipid peroxidation and ferroptosis (Bersuker et al., 2019).

It was found that the expression level of exosome miR-4443 in cisplatin resistant A549 NSCLC cells (A549-R) was higher than that in normal cells, and its expression level was 15 times higher than that in cisplatin sensitive A549 NSCLC cells (A549-S) (Song et al., 2021). Methyltransferase-like 3 (METTL3), a methyltransferase responsible for the methylation of N6 methyladenosine (m6A), has been found to be involved in the sensitivity of tumor cells to cisplatin and the proliferation and metastasis of NSCLC cells (Du et al., 2017; Jin et al., 2019; Li et al., 2021). Further studies showed that miR-4443 could directly target METTL3 and reduce its protein expression level, thereby reducing the content of m6A in cells. M6A sites were found at 2270, 2413, 4241, 4245 and 16006 in FSP1 mRNA, indicating that the expression of FSP1 may be regulated by m6A modification. In fact, in the cells transfected with miR-4443, the decrease of m6A enrichment of FSP1 and the up regulation of FSP1 mRNA expression were observed. In general, miR-4443 can reduce the expression of METTL3 at the protein level, thereby increasing the expression of FSP1, and regulate the sensitivity of NSCLC cells to cisplatin by regulating the ferroptosis pathway. In a mouse model of NSCLC xenografts, overexpression of miR-4443 rendered cisplatin treatment almost ineffective. Therefore, miR-4443 may be a target that sensitizes NSCLC cells to cisplatin. (Song et al., 2021).

In addition, it was found that circGFRA1 was significantly up-regulated in HER2 positive breast cancer, and silencing circGFRA1 could inhibit the proliferation, invasion and metastasis of HER2 positive breast cancer cells. In mechanism, circGFRA1 attenuated the inhibitory effect of miR-1228 on AIFM2 (FSP1) by adsorbing miR-1228. CircGFRA1/miR-1228/FSP1 axis participates in the development of HER2 positive breast cancer through ferroptosis pathway, and is a potential target for clinical treatment of HER2 positive breast cancer (Bazhabayi et al., 2021).

## MicroRNA targeting NRF2

Nuclear factor E2 related factor 2 (NRF2) is a transcription factor in cells and plays a very important role in the process of oxidative stress (Ma, 2013). Many enzymes or proteins involved in iron metabolism and lipid peroxidation are downstream target genes of NRF2 (Sun et al., 2016; Kerins and Ooi, 2018). It is known that ferritin (FTL/FTH1), ferroportin (FPN), heme oxygenase 1 (HMOX-1, HO-1), glutamic acid cysteine ligase (GCLC/GCLM), glutathione synthetase (GSS), NAD (P) H-dependent quinone oxidoreductase 1 (NQO1), SLC7A11 and GPX4 are all regulated by NRF2 (Anandhan et al., 2020; Wang et al., 2020; Hu et al., 2021). Under normal conditions, Kelch like ECH associated protein 1 (KEAP1) combines with cullin3 (CUL3) to form a ubiquitin E3 ligase complex, which ubiquitinates NRF2 and is rapidly degraded by proteasome. When under oxidative stress, the activity of ubiquitin E3 ligase complex decreased significantly, which hindered the ubiquitination and degradation of NRF2. The accumulated NRF2 can be transferred to the nucleus to form heterodimer with small muscle aponeurotic fibrosarcoma (sMAF) protein, and activate the transcription of genes containing antioxidant response elements (ARE). The expression of many genes is closely related to the inhibition of ferroptosis (Yamamoto et al., 2018).

MiR-6077 was found to be associated with cisplatin/pemetrexed (CDDP/PEM) resistance in patients with lung adenocarcinoma (LUAD). On the one hand, miR-6077 can inhibit (cyclin dependent kinase inhibitor 1A) CDKN1A, leading to the increase of (cyclin dependent kinase 1) CDK1, overcoming the block of G2/M point, thus enhancing the resistance of LUAD cells to CDDP/PEM; On the other hand, miR-6077 can inhibit KEAP1, activate NRF2/NQO1 signaling pathway, inhibit ferroptosis process, and enhance the resistance of LUAD cells to CDDP/PEM. In a mouse model of LUAD xenografts, overexpression of miR-6077 increased tumor size and inhibited the effect of CDDP/PEM therapy. This suggests that miR-6077 is a potential target for treating LUAD and increasing CDDP/PEM sensitivity. (Bi et al., 2022). In nasopharyngeal carcinoma (NPC), the significant down-regulation of Raf kinase inhibitor protein (RKIP) leads to the down-regulation of miR-450b-5p, thus activating NRF2/NQO1 signaling pathway and inhibiting ferroptosis process, which promotes NPC resistance to radiation (Huang et al., 2020). NQO1 is a widely distributed cytosolic flavoprotein, which can catalyze the double electron reduction of quinone compounds to hydroquinone in a NAD (P) H-dependent manner, avoiding the production of toxic semiquinone free radicals and reactive oxygen species (ROS), and preventing the direct reaction between quinone and intracellular sulfhydryl (Bianchet et al., 2008; Chhetri et al., 2018; Zhang Y. et al., 2018; Ross and Siegel, 2021).

MiR-130b-3p can inhibit the process of ferroptosis in melanoma treated with erastin or RSL3, and can reduce the content of lipid peroxide and Fe^2+^ in cells. In mechanism, miR-130b-3p inhibits ferroptosis by inhibiting the expression of Dikkopf associated protein 1 (DKK1) and activating NRF2/HO-1 signaling pathway (Liao et al., 2021).

It was found that the expression of lncRNA MT1DP could make A549 and H1299 NSCLC cells induced by erastin more sensitive to ferroptosis by down regulating NRF2. For tumor cells treated with erastin, lncRNA MT1DP can up regulate the contents of MDA and ROS, increase the concentration of Fe^2+^ and reduce the level of GSH. Through the study of its mechanism, lncRNA MT1DP inhibits the expression of NRF2 by stabilizing miR-365a-3p. Therefore, the lncRNA MT1DP/miR-365a-3p/NRF2 axis can be used as a new strategy to sensitize the ferroptosis of NSCLC cells induced by erastin (Gai et al., 2020).

## MicroRNA targeting glutamine metabolism

Glutamine can be introduced into cells by glutamine transporter (SLC1A5), and a part of glutamine is decomposed into glutamic acid by cytoplasmic GLS1, participating in the exchange of extracellular cystine and intracellular glutamic acid in system xc^−^. The other part enters mitochondria and is decomposed into glutamic acid under the catalysis of mitochondrial GLS2, and then is catalyzed by glutamic-oxaloacetic transaminase 1 (GOT1) to produce α- Ketoglutarate (α KG). α KG can participate in a series of reactions as the raw material of mitochondrial tricarboxylic acid cycle (TCA cycle) to promote the production of ROS (Hassannia et al., 2019). Inhibition of mitochondrial TCA cycle or electron transfer chain (ETC) can reduce mitochondrial membrane potential hyperpolarization and lipid peroxide accumulation, thus inhibiting the process of ferroptosis (Gao et al., 2019).

In melanoma cells, miR-137 inhibited SLC1A5, resulting in decreased glutamine uptake and MDA accumulation. Inhibition of miR-137 can increase the sensitivity of melanoma cells to ferroptosis induced by erastin or RSL3. This indicates that the miR-137/SLC1A5 axis can regulate the glutamine metabolic pathway, inhibit the participation of glutamine metabolites in the TCA cycle, thus reducing the production of ROS and the accumulation of lipid peroxides, and finally inhibit the occurrence of ferroptosis. In nude mice with melanoma xenografts, knockdown of miR-137 enhanced the effect of erastin and further reduced tumor volume. This further demonstrates the role of miR-137 in melanoma cells. (Luo et al., 2018).

In addition, overexpression of miR-9 in melanoma cells can directly bind to 3′-UTR of GOT1 and inhibit its activity, thus weakening ferroptosis induced by erastin or RSL3, while inhibition of miR-9 expression has the opposite result. Inhibition of lipid peroxidation and ferroptosis caused by miR-9 can be reversed by inhibiting glutamine transport and decomposition (Zhang et al., 2018b).

## Other targets

Activating transcription factor 4 (ATF4) is an important gene in the regulation of mitochondrial oxidative stress, which can activate the expression of SLC7A11 and other genes and play a role in anti-oxidative stress (Lewerenz et al., 2012). In the HepG2 and Hep3B hepatoma cells treated with erastin, the overexpression of miR-214-3p increased the level of MDA and ROS in the cells, increased the concentration of Fe^2+^, decreased the level of GSH, and increased the sensitivity of the cells to ferroptosis induced by erastin. This process was proved to be related to miR-214-3p directly targeting AFT4 and inhibiting its activity. When miR-214-overexpressing Hep3B hepatoma cells were subcutaneously injected into nude mice, it was found that the expression of miR-214 in tumor tissue was increased, while the expression of ATF4 was decreased. And miR-214 enhanced the effect of erastin, which further reduced tumor volume (Bai et al., 2020). In addition, it was found that lncRNA HULC plays a role as the ceRNA of miR-3200-5p in hepatocellular carcinoma, and miR-3200-5p can regulate the ferroptosis process by targeting ATF4 and inhibit the proliferation and metastasis of hepatoma cells (Guan et al., 2022).

Signal transducer and activator of transcription 3 (STAT3) is an important regulatory molecule in cells, which can regulate the expression of antioxidant stress genes such as SLC7A11 (Qiang et al., 2020), NRF2-GPX4 (Liu and Wang, 2019). In the study of the regulation of breast cancer cell proliferation, metastasis and ferroptosis by circular RNA RHOT1 (cirRHOT1), it was found that cirRHOT1 could adsorb miR-106a-5p and reduce its expression, weaken the inhibition of miR-106a-5p on STAT3, and finally enhance the resistance of MDA-MB-231 and T47D breast cancer cells to ferroptosis. In MDA-MB-231 cell xenografted nude mice, knockdown of cirRHOT1 can increase the expression of miR-106a-5p in tumor tissue, while reduce the expression of STAT3, and the tumor growth is significantly inhibited (Zhang et al., 2021a). In addition, propofol was found to inhibit the proliferation, invasion and metastasis of SGC7901 and BGC823 gastric cancer cells and promote the progress of ferroptosis. In terms of mechanism, propofol could up regulate miR-125b-5p and inhibit the expression of STAT3, promote the process of ferroptosis and inhibit the development of gastric cancer cells (Liu Y.P. et al., 2021).

GTP cyclohydrolase 1 (GCH1) was found to hinder the progression of ferroptosis. GCH1 can synthesize tetrahydrobiopterin/dihydrobiopterin (BH4/BH2) in cells and cause lipid remodeling. GCH1/BH4 axis is an antioxidant system independent of GSH/GPX4 axis, which can regulate the synthesis of BH4, the abundance of CoQ10 and prevent the peroxidation of phospholipids with two polyunsaturated fatty acyl tails, thus enhancing the ability of cells to resist ferroptosis (Kraft et al., 2020). In the exploration of bioinformatics, it was found that TMEM161B-AS1/hsa-miR-27a-3p/GCH1 regulatory network was involved in the development of esophageal cancer (Lu M. et al., 2022). As an independent antioxidant system in ferroptosis, GCH1/BH4 axis may have great potential for the regulation and progress of ferroptosis, which requires further exploration by researchers.

OTU domain-containing ubiquitin aldehyde-binding protein 1 (OTUB1) was found to be overexpressed in human tumors. OTUB1 can be directly combined with SLC7A11 to make it stable. CD44 also plays an important role in this process, which can promote the interaction between OTUB1 and SLC7A11 and enhance the stability of SLC7A11. Knockout of OTUB1 will reduce the expression of SLC7A11, and knockout of CD44 will weaken the interaction between OTUB1 and SLC7A11, both of which regulate the progress of ferroptosis by affecting the stability of SLC7A11 (Liu et al., 2019).

Ferroptosis regulation is a complex system, and many regulatory factors play a crucial role in the progress of ferroptosis. Ser90/93/96 of beclin1(BECN1) can phosphorylate under the action of AMP activated protein kinase (AMPK), and then form a complex with SLC7A11 to inhibit system xc^−^ activity and promote the progress of ferroptosis (Song et al., 2018). BRCA1 related protein 1 (BAP1) can encode nuclear deubiquitinating enzyme, which reduce histone 2A ubiquitination (H2AUB) on chromatin. BAP1 acts on SLC7A11 to reduce the H2AUB ratio of its promoter, and inhibits the expression of SLC7A11 through deubiquitination (Zhang Y. et al., 2018). Ataxia telangiectasia-mutated gene (ATM) silencing can enhance the nuclear transfer of metal regulated transcription factor 1 (MTF1), promote the expression of ferritin (FTH1, FTL) and ferroportin (FPN), and inhibit the progress of ferroptosis (Chen et al., 2020). In addition, P53 and Yes associated protein 1 (YAP1) (Ye et al., 2021) are closely related to the progress of ferroptosis. These targets are potential targets of miRNA, which have very important implications for us to explore the mechanism of miRNA acting on tumors and find targets for tumor treatment.

## Application and effect in treatment

With the development of research, more and more miRNAs have been found to regulate tumor development through ferroptosis pathway. This provides many potential targets for the treatment of tumors and the improvement of drug resistance.

It was found that lidocaine can promote ferroptosis of ovarian cancer and breast cancer cells and inhibit their proliferation *in vitro* by targeting miR-382-5p/SLC7A11 axis. *In vivo* experiments of animals, the expression of miR-382-5p was up-regulated and the expression of SLC7A11 was down-regulated in the ovarian cancer transplanted tumor bearing mice treated with lidocaine, and the tumor volume was significantly smaller than that of the control group nude mice (Sun D. et al., 2021). In conclusion, lidocaine has been found to have the potential to treat breast cancer and ovarian cancer by inducing ferroptosis of tumor cells. However, many problems need to be considered before it is applied in clinical practice. Lidocaine has many adverse reactions, which may cause central nervous system reactions, such as paresthesia, muscle tremor, convulsion, respiratory depression, etc., and may also cause cardiovascular reactions, such as hypotension, bradycardia, atrioventricular block, etc. Therefore, the efficacy and adverse reactions of lidocaine need to be further evaluated in the clinic.

Propofol is a commonly used anesthetic in clinic. It has been found that it has a good inhibitory effect on the proliferation, invasion and migration of gastric cancer cells, and can increase the effect of erastin. Propofol can promote ferroptosis of gastric cancer cells by targeting miR-125b-5p/STAT3 axis, and the tumor volume of gastric cancer transplanted tumor bearing mice treated with propofol has been well controlled (Liu Y.P. et al., 2021). Ketamine is an intravenous anesthetic with sedative, analgesic and anesthetic effects, which is used for various minor operations or diagnostic operations. Recent studies have found that ketamine can promote the ferroptosis of liver cancer cells by downregulating lncRNA PVT1. As a ceRNA, downregulation of lncRNA PVT1 reduced the adsorption of miR-214-3p, while increased the binding of miR-214-3p to GPX4 (He et al., 2021). Although both drugs were found to induce ferroptosis in specific tumors, they are unlikely to be approved for clinical use in tumors due to concerns over abuse.

Metformin is a common hypoglycemic drug. It was found to inhibit the proliferation of breast cancer cells by targeting miR-324-3p/GPX4 axis. In breast cancer transplanted tumor bearing mice treated with metformin, the expression of miR-324-3p and GPX4 were up-regulated and down-regulated respectively, and the tumor volume was significantly smaller than that of the control mice (Hou et al., 2021). Although metformin has the effect of anti-breast cancer, the hypoglycemic effect needs to be noticed. It can reduce the blood sugar content, cause hypoglycemia in patients, and cause other diseases. This problem may be solved by combining with other drugs and supplementing glucose. In addition, for breast cancer patients with diabetes, this may be a good adjuvant.

In addition to some chemical synthetic drugs, some natural products have also been found to be able to induce ferroptosis in tumor cells by targeting ferroptosis related miRNAs. Icariside II (ICS II) is an active flavonoid with anti-tumor properties. It can promote the ferroptosis of RCC cells by targeting miR-324-3p/GPX4 axis, which is independent of p53. ICs II can inhibit the proliferation, invasion and migration of RCC cells, but has no obvious effect on the viability of normal cells (Yu et al., 2022). Curcumenol is an effective component of wenyujin, which has anti-tumor effect. Ferroptosis is the main form of curcumenol induced lung cancer cells death, which has been proved *in vitro* and *in vivo* experiments. The mechanism is that curcumenol can target the lncRNA H19/miR-19b-3p/FTH1 pathway (Zhang R. et al., 2022).

Although both chemical synthetic drugs and natural products have the ability to target miRNAs to promote ferroptosis in tumor cells, they have some common shortcomings. First, the mechanism by which drugs regulate miRNAs or related molecules is unclear. Second, there are many targets of drug regulation, resulting in poor specificity and many side effects. In order to accurately target the target molecules, the research of genetic drugs will become a hotspot in the future.

In a previous report, programmed death receptor 1 (PD-1) antibody (anti-PD-1) was able to reactivate tumor infiltrating CD8 + T cells in the TME, which released interferon- γ (IFN- γ) that can promote the ferroptosis of tumor cells. Anti-PD-1 immunotherapy for melanoma transplanted mice can inhibit tumor growth and increase the content of lipid ROS and ferroptosis marker prostaglandin-endoperoxide synthase 2 (PTGS2). This result was blocked by ferroptosis inhibitor liproxstatin-1, which indicates that ferroptosis is one of the important mechanisms of anti-PD-1 immunotherapy for melanoma. In immunotherapy, IFN- γ is the main effector that triggers ferroptosis in tumor cells. Guo et al. (2022) found that one of the important mechanisms for ferroptosis induced by IFN- γ in melanoma is the activation of the ATF3/miR-21-3p/TXNRD1 axis, and blocking this axis attenuates ferroptosis and therapeutic effect of IFN- γ, which indicates that the overexpression of miR-21-3p may play a synergistic role with anti-PD-1 immunotherapy. In order to verify whether miR-21-3p can enhance the effect of anti-PD-1 *in vivo*, the researchers constructed miR-21-3p-loaded gold nanoparticles (miR-21-3p-AuNp), injected them into melanoma transplanted mice, and injected anti-PD-1 for immunotherapy. It was found that compared with mice treated with miR-21-3p-AuNp or anti-PD-1 alone, the tumor growth of mice treated with the combination was further delayed, and the contents of miR-21-3p and TXNRD1 in tumor tissue were further up-regulated and down-regulated respectively, and the content of ferroptosis marker PTGS2 was higher. This result can be blocked by the ferroptosis inhibitor liproxstatin-1. In addition, gold nanoparticles (AuNp) alone had no therapeutic effect on tumors, and the therapeutic effect of miR-21-3p-AuNp was attributed to miR-21-3p. This indicates that miR-21-3p can enhance ferroptosis induced by anti-PD-1 immunotherapy. In addition, the combined treatment of miR-21-3p-AuNp and anti-PD-1 can promote the expression and secretion of pro-inflammatory cytokines and chemokines, thereby enhancing anti-tumor immunity. Excitingly, the miR-21-3p-AuNp delivery system has low immunogenicity. Moreover, the liver, spleen, kidney, heart and lung of the mice did not show obvious pathological changes in the results of HE staining. This shows that miR-21-3p-AuNp treatment has relatively high safety and has the potential to enter clinical treatment.

Luo et al. determined that miR-101-3p/TBLR1 axis can promote ferroptosis and apoptosis of lung cancer cells and significantly inhibit cell proliferation *in vitro* experiments. They combined miR-101-3p with nanocarriers to make nano drugs, and proved that they could be well clustered in the tumor tissues of lung cancer bearing mice through fluorescence labeling experiments. In the model mice injected with nano drugs through tail vein, the expression of miR-101-3p and TBLR1 in tumor tissue increased and decreased respectively, and some molecules were significantly changed, such as the content of ROS and lipid ROS increased, the content of GSH decreased, the ferroptosis markers GPX4 and PTGS2 decreased and increased respectively, the apoptosis markers Bcl-2 and cleaved Caspase3 decreased and increased respectively, and the tumor tissue volume was significantly smaller than that of the control group (Luo Y. et al., 2021). This indicates that nano drugs can play a good anti-tumor effect by promoting ferroptosis and apoptosis of tumor tissues in mice.

In conclusion, compared with traditional drugs, genetic drugs have higher specificity. However, it should also be noted that miRNAs are pleiotropic and can target many downstream genes to produce different biological effects, such as the regulation of cell cycle, apoptosis, pyroptosis, ferroptosis, and tumor immunity. Therefore, it is very important to deeply study the specific mechanism of miRNA, which provides a theoretical basis for gene therapy. In addition, it is important to accurately transfer specific miRNAs to specific tumor sites. Although in the study of Guo et al., miR-21-3p hardly caused damage to other organs and had no obvious immunogenicity, it was only tested in short-term experiments and could not predict the adverse effects of long-term use. In addition, it is more unclear whether other miRNAs will cause adverse reactions. As far as ferroptosis related miRNAs are concerned, promoting ferroptosis of tumor cells can inhibit tumors, while promoting ferroptosis of central nervous cells and other normal organs may lead to neurodegenerative diseases, organ damage and organ fibrosis. Therefore, how to transfer miRNAs specifically into specific tumors requires further exploration by researchers, and multidisciplinary cross fusion will be beneficial to this process.

## Conclusions and prospects

Malignant tumor is a disease that seriously endangers human life and health. At present, there are surgical resection, chemotherapy, radiotherapy and immunotherapy. However, the mechanism of tumor occurrence and development is very complex. Tumor has strong resistance to some traditional treatment methods and maintains a high recurrence rate. Therefore, the mortality of tumor patients is still very high. Individualized treatment will be the basic direction of tumor treatment in the future. Only by understanding the molecular mechanism of tumor development in patients and the differential expression information of related genes, can we better improve the treatment effect and improve the survival rate of patients. MicroRNA (miRNA) is a kind of small non coding RNA, which can directly combine with the 3′- untranslated region (3' - UTR) of the target mRNA to inhibit the translation of the target mRNA, thus regulating a series of cellular biological processes. MiRNA has been found to play a key role in the development of tumors. The differential expression of miRNA is closely related to the degree of malignancy, drug resistance and prognosis of tumors. Regulating the expression of miRNAs can inhibit or kill tumor cells through a variety of mechanisms, including inhibiting cell cycle (Zhang Z. et al., 2018), inhibiting stem cell frequency (Ni et al., 2021), inhibiting angiogenesis (Lu et al., 2017), promoting tumor immunity (Wang C. et al., 2021), promoting apoptosis (Xu W. et al., 2021), pyroptosis (Jiang et al., 2020), ferroptosis (Luo et al., 2018), etc. Ferroptosis is a form of cell death discovered in recent years. It is a kind of programmed death caused by iron dependent lipid peroxidation, which is different from apoptosis, necrosis and other cell death modes. The discovery of new cell death modes has great significance for the treatment of tumor diseases. More and more studies have found that miRNA regulates tumor progression by regulating ferroptosis signaling pathway. Therefore, this paper expounds the mechanism of ferroptosis, and summarizes the research progress of miRNA targeting ferroptosis signaling pathway in tumors.

**FIGURE 1:**
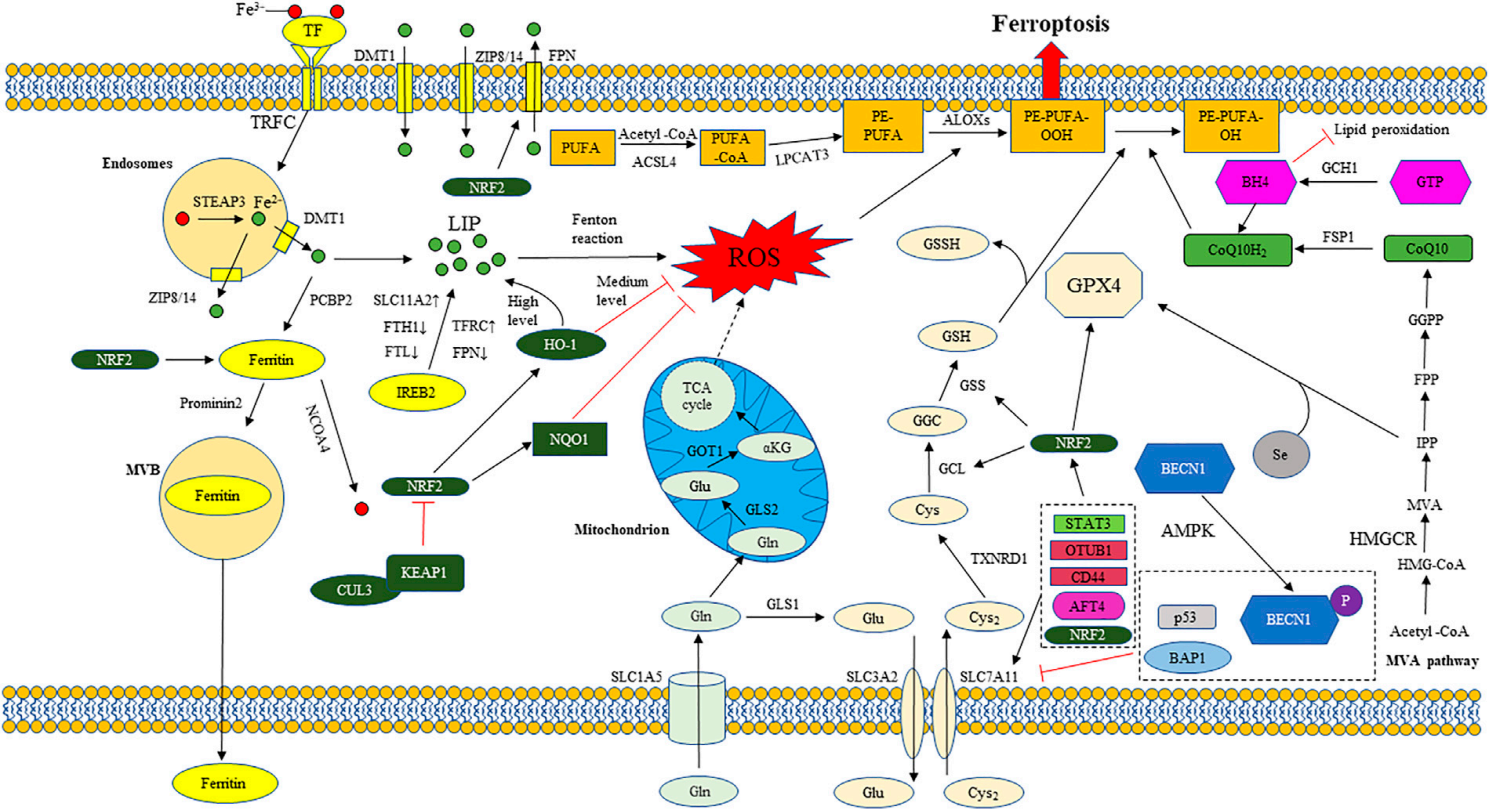
The regulatory mechanism of ferroptosis. The regulatory network of ferroptosis is mainly divided into two categories: Oxidation system and antioxidant system. The oxidation system includes iron metabolism, lipid metabolism, glutamate metabolism, etc. The antioxidant system includes system xc^−^/GSH/GPX4 axis, FSP1/CoQ10/NAD (P) H axis, GCH1/BH4 axis, etc. Iron metabolism: Transferrin (TF); Transferrin receptor 1 (TFRC); Six transmembrane epithelial antigen of protein 3 (STEAP3); Divalent metal ion transporter 1 (DMT1); Zinc transporter 8/14 (ZIP8/14); Labile iron pool (LIP); ferroportin (FPN); Ferritin heavy chain 1 (FTH1); Ferritin light chain (FTL); Solute carrier family member 11A2 (SLC11A2); Iron response element binding protein 2 (IREB2); Reactive oxygen species (ROS); Poly (RC) binding protein 2 (PCBP2); Nuclear receptor coactivator 4 (NCOA4); Multivesicular bodies (MVB). Lipid metabolism: Polyunsaturated fatty acid (PUFA); Long chain acyl CoA synthetase 4 (ACSL4); Lysophosphatidylcholine acyltransferase 3 (LPCAT3); Phosphatidylethanolamine (PE); Arachidonic acid lipid peroxidase (ALOX). Glutamate metabolism: Glutamine transporter (SLC1A5); Glutamic acid cysteine ligase 1 (GCL1); Glutamic acid cysteine ligase 2 (GCL2); Glutamic-oxaloacetic transaminase 1 (GOT1); α- Ketoglutarate (α KG). Tricarboxylic acid cycle (TCA cycle). System xc^-^/GSH/GPX4 axis: Cystine/glutamate antiporter (system xc^−^); Solute carrier family member 7A11 (SLC7A11); Solute carrier family member 3A2 (SLC3A2); Glutathione (GSH); Thioredoxin reductase 1 (TXNRD1); Glutamic acid cysteine ligase (GCL); Glutathione synthetase (GSS); Glutathione peroxidase 4 (GPX4); oxidized glutathione (GSSG); Selenium (Se). FSP1/CoQ10/NAD (P) H axis: ferroptosis suppressor protein 1 (FSP1); Oxidized coenzyme Q10 (CoQ10); Reduced coenzyme Q10 (CoQ10H_2_). 3-hydroxy-3-methyl glutaryl coenzyme A (HMG-CoA); 3-hydroxy-3-methyl glutaryl coenzyme A reductase (HMGCR); Mevalonate (MVA); Isopentenyl pyrophosphate (IPP); Farnesyl pyrophosphate (FPP); Geranylgeranyl pyrophosphate (GGPP). GCH1/BH4 axis: Guanosine triphosphate (GTP); GTP cyclohydrolase 1 (GCH1); Tetrahydrobiopterin (BH4); Other regulatory factors: Nuclear factor E2 related factor 2 (NRF2); Heme oxygenase 1 (HO-1); NAD (P) H-dependent quinone oxidoreductase 1 (NQO1); Kelch like ECH associated protein 1 (KEAP1); Cullin3 (CUL3); Activating transcription factor 4 (ATF4); Activating transcription factor 4 (ATF4); OTU domain-containing ubiquitin aldehyde-binding protein 1 (OTUB1); Beclin1(BECN1); AMP activated protein kinase (AMPK); BRCA1 related protein 1 (BAP1); Histone 2A ubiquitination (H2AUB); Yes associated protein 1 (YAP1); Signal transducer and activator of transcription 3 (STAT3).

The regulatory network of ferroptosis is mainly divided into two categories: oxidation system and antioxidant system. The oxidation system includes iron metabolism, lipid metabolism, glutamate metabolism, etc. in iron metabolism, the related targets that regulate the concentration of free iron in cells affect the progress of ferroptosis. The increased expression of TF, TFRC, STEAP3, DMT1, NCOA4 and IREB2 contribute to the increase of intracellular free iron concentration, which can promote the progress of ferroptosis. While the increased expression of FPN, FTH1, FTL and prominin 2 attenuate the effect of ferroptosis. In lipid metabolism, the content of PUFA in the plasma membrane determines the sensitivity of cell ferroptosis, because PUFA is the main substrate of lipid peroxidation. The enhanced expression of ACSL4 and LPCAT3, which are involved in the induction of PUFA transfer to the plasma membrane, can promote the progress of ferroptosis. The increased expression of ALOXs can directly promote lipid peroxidation and ALOXs are the direct regulator of ferroptosis. Glutamate metabolic pathway can produce α KG, α Kg is the raw material of mitochondrial TCA cycle, which can promote the mitochondrial TCA cycle to produce ROS, which contributes to the promotion of ferroptosis. Therefore, increasing the expression of SLC1A5, GLS2 and GOT1 in glutamate pathway can promote the generation of ROS and the progress of ferroptosis. The antioxidant system includes system xc^−^/GSH/GPX4 axis, FSP1/CoQ10/NAD (P) H axis, GCH1/BH4 axis, etc. If the activities of SLC7A11, GPX4 and GSH synthesis pathway related enzymes are inhibited, the antioxidant capacity of system xc^−^/GSH/GPX4 axis will be destroyed, and the lipid peroxide in cells cannot be removed in time, resulting in ferroptosis. If FSP1 is inhibited, the reduced CoQ10 will be reduced, and the scavenging capacity of intracellular lipid peroxide will be decreased, which will promote the progress of ferroptosis. GCH1 can catalyze the hydrolysis of GTP to BH4, increase the abundance of CoQ10 and prevent the peroxidation of phospholipids with two polyunsaturated fatty acyl tails. If GCH1 is inhibited, the ability of cells to resist lipid peroxidation will be weakened and the development of ferroptosis will be accelerated. In addition, NRF2, ATF4, STAT3, BECN1, BAP1, ATM, P53, YAP1, OTUB1, CD44 and other genes also participate in the progress of ferroptosis by regulating the related targets of oxidation system and antioxidant system (Figure 1). It is worth noting that some factors involved in the regulation of ferroptosis have two sides. For example, in different studies, the effect of HO-1 on ferroptosis shows opposite results (Ma et al., 2020; Chen Y.et al., 2021; Liu X.J. et al., 2021; Yang J. et al., 2021). HO-1 is a stress-induced enzyme that can catalyze the degradation of heme to produce biliverdin, carbon monoxide (CO) and Fe^2+^ (Pulkkinen et al., 2011). Biliverdin and CO have strong antioxidant capacity, which can reduce the content of ROS and inhibit lipid peroxidation. The production of Fe^2+^ can promote the production of ferritin and ferritin can bind free Fe^2+^. But the excessive increase of Fe^2+^ concentration will lead to a large increase in ROS and lipid peroxidation, significantly promoting the progress of ferroptosis. Therefore, the mechanism of HO-1 is complex (Wang et al., 2007; Sugimoto et al., 2012; Chiang et al., 2018). The effect of HO-1 on the progress of ferroptosis may be related to its specific expression level in cells. At the medium level, HO-1 shows antioxidant effect, while at the high level, it can significantly increase the concentration of Fe^2+^ and promote ferroptosis (Chiang et al., 2018). MiRNAs targeting these ferroptosis targets have been found in different tumor studies, which provides a theoretical basis for the treatment of tumors by miRNAs (Table 1).

**TABLE 1:** The basic information of miRNA in ferroptosis signaling pathway.

miRNA	Research object	Target	Progress on ferroptosis	References
miR-545	Colorectal cancer	TF	Inhibition	Zheng et al. (2021)
miR-19a	Colorectal cancer	IREB2	Inhibition	Fan et al. (2022)
miR-4735-3p	Clear cell renal cell carcinoma	FPN	Promotion	Zhu et al. (2022)
miR-129-5p	Bladder cancer	PROM2	Promotion	Luo W. et al. (2021)
miR-19b-3p	Lung cancer	FTH1	Inhibition	Zhang R. et al. (2022)
miR-375	Gastric cancer	SLC7A11	Promotion	Ni et al. (2021)
miR-5096	Breast cancer	SLC7A11	Promotion	Yadav et al. (2021)
miR-382-5p	Ovarian and breast cancer	SLC7A11	Promotion	Sun D. et al. (2021)
miR-489-5p	Gastric cancer	SLC7A11	Promotion	Mao et al. (2021)
miR-545-3p	Thyroid cancer	SLC7A11	Promotion	Wang H.H. et al. (2021)
miR-125b-5p	Oral squamous cell carcinomas	SLC7A11	Promotion	Yu et al. (2021)
miR-34c-3p	Oral squamous cell carcinomas	SLC7A11	Promotion	Sun et al. (2022)
miR-375-3p	Cervical cancer	SLC7A11	Promotion	Wu et al. (2021)
miR-409-3p	Cervical cancer	SLC7A11	Promotion	Wu et al. (2021)
miR-515-5p	Cervical cancer	SLC7A11	Promotion	Wu et al. (2021)
miR-1261	Hepatocellular carcinoma	SLC7A11	Promotion	Lyu et al. (2021)
miR-25-3p	Prostate cancer	SLC7A11	Promotion	Jiang et al. (2022)
miR-143-3p	Renal cell carcinoma	SLC7A11	Promotion	Li et al. (2022)
miR-15a-3p	Colorectal cancer	GPX4	Promotion	Liu et al. (2022)
miR-539	Colorectal cancer	GPX4	Promotion	Yang Y. et al. (2021)
miR-324-3p	Lung adenocarcinoma	GPX4	Promotion	Deng et al. (2021)
miR-324-3p	Breast cancer	GPX4	Promotion	Hou et al. (2021)
miR-214-3p	Liver cancer	GPX4	Promotion	He et al. (2021)
miR-1287-5p	Osteosarcoma	GPX4	Promotion	Xu Z. et al. (2021)
miR-1231	Papillary thyroid cancer	GPX4	Promotion	Chen W.et al. (2021)
miR-15a	Prostate cancer	GPX4	Promotion	Xu et al. (2022)
miR-541-3p	Hepatocellular carcinoma	GPX4	Promotion	Xu et al. (2020)
miR-1287-5p	Non-small cell lung cancer	GPX4	Promotion	Shanshan et al. (2021)
miR-522	Gastric cancer	ALOX15	Inhibition	Zhang et al. (2020)
miR-7-5p	Radioresistant HeLa and SAS cell lines	ALOX12	Inhibition	Tomita et al. (2021)
miR-18a	Glioblastoma	ALOXE3	Inhibition	Yang X. et al. (2021)
miR-23a-3p	Hepatocellular carcinoma	ACSL4	Inhibition	Lu Y. et al. (2022)
miR-424-5p	Ovarian cancer	ACSL4	Inhibition	Ma et al. (2021)
miR-670-3p	Glioblastoma	ACSL4	Inhibition	Bao et al. (2021)
miR-4291	Cervical cancer	ACSL4	Inhibition	Ou et al. (2022)
miR-4443	Non-small cell lung carcinoma	FSP1	Inhibition	Song et al. (2021)
miR-1228	HER-2-positive breast cancer	FSP1	Inhibition	Bazhabayi et al. (2021)
miR-6077	Lung adenocarcinoma	NRF2	Promotion	Bi et al. (2022)
miR-450b-5p	Nasopharyngeal carcinoma	NRF2	Promotion	Huang et al. (2020)
miR-130b-3p	Melanoma	NRF2	Inhibition	Liao et al. (2021)
miR-365a-3p	Non-small cell lung cancer	NRF2	Promotion	Gai et al. (2020)
miR-137	Melanoma	SLC1A5	Inhibition	Luo et al. (2018)
miR-9	Melanoma	GOT1	Inhibition	Zhang et al. (2018a)
miR-214-5p	Hepatoma	ATF4	Promotion	Bai et al. (2020)
miR-3200-5p	Hepatocellular carcinoma	ATF4	Promotion	Guan et al. (2022)
miR-106a-5p	Breast cancer	STAT3	Promotion	Zhang et al. (2021a)
miR-125b-5p	Gastric cancer	STAT3	Promotion	Liu Y.P. et al. (2021)
miR-27a-3p	Esophageal cancer	GCH1	Promotion	Lu M. et al. (2022)
miR-101-3p	Lung cancer	TBLR1	Promotion	Luo Y. et al. (2021)
miR-21-3p	Melanoma	TXNRD1	Promotion	Luo Y. et al. (2021)

This table collates the relationship between miRNAs and ferroptosis targets in different tumors and their promoting or inhibiting effects on ferroptosis process.

TF, Transferrin; IREB2, Iron response element binding protein 2; FPN, ferroportin; PROM2, Prominin2; FTH1, Ferritin heavy chain 1; SLC7A11, Solute carrier family member 7A11; GPX4, Glutathione peroxidase 4; ALOX15, Arachidonic acid lipid peroxidase 15; ALOX12, Arachidonic acid lipid peroxidase 12; ALOXE3, Arachidonic acid lipid peroxidase E3; ACSL4, Long chain acyl CoA synthetase 4; FSP1, ferroptosis suppressor protein 1; NRF2, Nuclear factor E2 related factor 2; SLC1A5, Glutamine transporter; GOT1, Glutamic-oxaloacetic transaminase 1; ATF4, Activating transcription factor 4; STAT3, Signal transducer and activator of transcription 3; GCH1, GTP cyclohydrolase 1; TBLR1, Transducin beta 1X-linked receptor protein 1; TXNRD1, Thioredoxin reductase 1.

Finally, we discuss the application of therapies targeting ferroptosis associated miRNAs. Some chemical synthetic drugs and natural products were found to be able to target ferroptosis related miRNAs for tumor therapy, but this class of drugs has some common inadequacies. First, the mechanisms by which chemical synthetic drugs and natural products regulate miRNAs or related molecules are unknown. Second, chemical synthetic drugs and natural products can target many targets, resulting in poor specificity and many side reactions. Therefore, gene drugs with high specificity are hot spots for future research. Currently, gene drugs for ferroptosis related miRNAs have been preliminarily explored and the effects have been validated in animals. For example, gold nanoparticles loaded with miR-21-3p to treat melanoma tumor bearing mice were able to render them more sensitive to anti-PD-1 immunotherapy by promoting ferroptosis. Nano drugs loaded with miR-101-3p can play a good anti-tumor effect by promoting ferroptosis and apoptosis of tumor tissues in mice. The use of ferroptosis inhibitors can block the ferroptosis and tumor treatment effects induced by the above-mentioned gene drugs, which suggests that ferroptosis plays a great role in these miRNA treatments. However, the role of miRNA is pleiotropic, and it can participate in a series of biological processes such as cell cycle, cell apoptosis, ferroptosis, and tumor immunity. Therefore, ferroptosis is only one effect of miRNA, and the anti-tumor effect of miRNA is a combination of multiple mechanisms. For example, miR-101-3p can induce lung cancer cell death through apoptosis and ferroptosis. As another example, miR-6077 affects the sensitivity of LUAD cells to CDDP/PEM by regulating cell cycle genes and ferroptosis.

Therefore, in-depth study of the mechanism of miRNA is the theoretical basis of miRNA gene drug therapy. The in-depth study of the mechanism of miRNA is helpful for us to master the function of a miRNA in a specific tumor. In addition, how to specifically transfer miRNAs into specific tumors is a key issue, because miRNAs play different roles in different tissues and may cause some side effects. For example, miR-375-3p can promote ferroptosis of cervical cancer cells by targeting SLC7A11, and can be used as a potential gene therapy drug. However, Zhang et al. found that miR-375-3p could induce ferroptosis in cardiomyocytes by targeting GPX4, thus promoting fibrosis (Zhuang et al., 2022). For another example, miR-214-3p can inhibit tumor by targeting GPX4 to induce ferroptosis in liver cancer cells, while in another study, miR-214-3p can aggravate ferroptosis in cisplatin induced acute kidney injury by targeting GPX4 (Zhou et al., 2022). This suggests that ferroptosis related miRNAs may increase the risk of organ damage and fibrosis in non-tumor tissues. Therefore, how to transfer miRNAs specifically into specific tumors requires further exploration by researchers, and multidisciplinary cross fusion will be beneficial to this process.

In conclusion, ferroptosis is a complex regulatory network, which is different from the death forms of apoptosis and necrosis. It is a new idea for the treatment of anti-apoptotic tumor cells. In the past, the role of miRNA in tumors was often associated with cell cycle regulatory genes, but in recent years, it has been found that some miRNAs can regulate the occurrence and development of tumors through the ferroptosis pathway. This is of great significance for understanding the role of miRNA in tumors, finding therapeutic targets and improving drug sensitivity.

## Glossary

miRNA: MicroRNA
3′—UTR: 3′—untranslated region
TF: transferrin
TFR1/TFRC: transferrin receptor 1
STEAP3: six transmembrane epithelial of antigen protein 3
DMT1/SLC11A2: divalent metal ion transporter 1
ZIP8: zinc transporter 8
ZIP14: zinc transporter 14
LIP: labile iron pool
ROS: reactive oxygen species
PCBP1: poly (RC) binding protein 1
PCBP2: poly (RC) binding protein 2
NCOA4: nuclear receptor coactivator 4
IREB2: iron response element binding protein 2
IRE: iron response element
FTH1: ferritin heavy chain 1
FTL: ferritin light chain
FPN/SLC40A1: ferroportin
MVB: multivesicular bodies
CRC: colorectal cancer
MDA: malondialdehyde
ccRCC: clear cell renal cell carcinoma
lncRNA: long-chain noncoding RNA
circRNA: circular RNA
ceRNA: competitive endogenous RNA
system xc-: cystine/glutamate antiporter
SLC7A11: solute carrier family member 7A11
SLC3A2: solute carrier family member 3A2
GLS: glutaminase
GLS1: glutaminase 1
GLS2: glutaminase 2
GSH: glutathione
GCL: glutamic acid cysteine ligase
GSS: glutathione synthetase
GPX4: glutathione peroxidase 4
GC: gastric cancer
TNFAIP8/TIP8: tumor necrosis factor (TNF)- α Induced protein 8
NSCLC: non-small cell lung cancer
PUFA: polyunsaturated fatty acid
MUFA: monounsaturated fatty acids
AA: arachidonic acid
AdA: adrenal acid
ACSL4: long chain acyl CoA synthetase 4
LPCAT3: lysophosphatidylcholinyltransferase 3
PE: phosphatidylethanolamine
LOX: lipoxygenase
MDA: malondialdehyde
4-HNEs: 4-hydroxy-nonanal
EV: extracellular vesicle
CAFs: cancer associated fibroblasts
TME: tumor microenvironment
ALOX15: arachidonic acid lipoxygenase 15
hnRNPA1: heterogeneous nuclear ribonucleoprotein A1
USP7: ubiquitin specific protease 7
CRR: clinically relevant anti-radiation
GBM: glioblastoma
ALOXE3: arachidonic acid lipoxygenase E3
12-HETE: 12-hydroxyeicosapentaenoic acid
GsPCR: Gs protein coupled receptor
ETS1: ETS proto-oncogene 1
AIFM2: mitochondrial apoptosis inducing factor 2
FSP1: ferroptosis suppressor protein 1
CoQ: coenzyme Q
MVA: mevalonate
HMG CoA: 3-hydroxy-3-methylglutaric acid monoacyl CoA
HMGCR: HMG CoA reductase
IPP: isopentenyl pyrophosphate
FPP: farnesyl pyrophosphate
GGPP: geranylgeranyl pyrophosphate
A549-R: cisplatin resistant A549 NSCLC cells
A549-S: cisplatin sensitive A549 NSCLC cells
METTL3: methyltransferase-like 3
m6A: N6 methyladenosine
NRF2: nuclear factor E2 related factor 2
HMOX-1/HO-1: heme oxygenase 1
GCLC: glutamic acid cysteine ligase catalytic subunit
GCLM: glutamic acid cysteine ligase regulatory subunit
NQO1: NAD (P) H-dependent quinone oxidoreductase 1
KEAP1: Kelch like ECH associated protein 1
CUL3: combines with cullin3
sMAF: small muscle aponeurotic fibrosarcoma
ARE: antioxidant response elements
CDDP: cisplatin
PEM: pemetrexed
LUAD: lung adenocarcinoma
CDKN1A: cyclin dependent kinase inhibitor 1A
CDK1: cyclin dependent kinase 1
NPC: nasopharyngeal carcinoma
RKIP: Raf kinase inhibitor protein
SLC1A5: glutamine transporter
GOT1: glutamic-oxaloacetic transaminase 1
α KG: α- Ketoglutarate
TCA cycle: tricarboxylic acid cycle
ETC: electron transfer chain
ATF4: activating transcription factor 4
STAT3: signal transducer and activator of transcription 3
GCH1: GTP cyclohydrolase 1
BH4: tetrahydrobiopterin
BH2: dihydrobiopterin
OTUB1: OTU domain-containing ubiquitin aldehyde-binding protein 1
BECN1: beclin1
AMPK: AMP activated protein kinase
BAP1: BRCA1 related protein 1
H2AUB: histone 2A ubiquitination
ATM: ataxia telangiectasia-mutated gene
MTF1: metal regulated transcription factor 1
YAP1: Yes associated protein 1
CO: carbon monoxide
TBLR1: transducin beta 1X-linked receptor protein 1
PTGS2: prostaglandin endoperoxide synthase 2
TXNRD1: thioredoxin reductase 1.
